# Validity of Self-Reported Weight and Height of Adolescents, Its Impact on Classification into BMI-Categories and the Association with Weighing Behaviour

**DOI:** 10.3390/ijerph6102696

**Published:** 2009-10-20

**Authors:** Tineke De Vriendt, Inge Huybrechts, Charlene Ottevaere, Inge Van Trimpont, Stefaan De Henauw

**Affiliations:** 1 Department of Public Health, Faculty of Medicine and Health Sciences, Ghent University, De Pintelaan 185, 2 blok A, B-9000 Ghent, Belgium; E-Mails: inge.huybrechts@ugent.be (I.H.); charlene.ottevaere@ugent.be (C.O.); 2 Research Foundation–Flanders, Egmontstraat 5, B-1000 Brussels, Belgium; 3 Centre for Pupils Counselling (CLB), Flemish Community Education, Jan Verspeyenstraat 3, B-9000 Ghent, Belgium; E-Mail: inge.van.trimpont@g-o.be; 4 Department of Health Sciences, Vesalius, Hogeschool Gent, Keramiekstraat 80, B-9000 Ghent, Belgium; E-Mail: stefaan.dehenauw@ugent.be

**Keywords:** height, weight, body mass index, validity, adolescents, weighing behaviour

## Abstract

This paper investigated the validity of self-reported height and weight of adolescents for the diagnosis of underweight, overweight and obesity and the influence of weighing behaviour on the accuracy. A total of 982 adolescents reported their height, weight, weighing behaviour and eating patterns in a questionnaire. Afterwards, their height and weight were measured and their Body Mass Index (BMI)-categories were determined using age- and gender-specific BMI cut-off points. Both girls and boys underreported their weight, whilst height was overestimated by girls and underestimated by boys. Cohen’s d indicated that these misreportings were in fact trivial. The prevalence of underweight was overestimated when using the self-reported BMI for classification, whilst the prevalence of overweight and obesity was underestimated. Gender and educational level influenced the accuracy of the adolescents’ self-reported BMI. Weighing behaviour only positively influenced the accuracy of the self-reported weight and not height or BMI. In summary, adolescents’ self-reported weight and height cannot replace measured values to determine their BMI-category, and thus the latter are highly recommended when investigating underweight, overweight and obesity in adolescents.

## 1. Introduction

The aetiology of overweight and obesity has its origins in childhood and adolescence and it has become an increasingly epidemic problem in young children and adolescents worldwide [1]. A close monitoring of height and weight in children and adolescents is necessary to detect any tendencies in the prevalence of overweight and obesity. In this context, valid weight and height data are critical, but measuring these anthropometrics is not always feasible due to logistic or budgetary limitations. Therefore, these data are in practice often self- or proxy-reported (by parents). The validity of self-reported weight and height has already been studied in adults, demonstrating a high correlation between self-reported and measured weight, height and body mass index [BMI, defined as weight (kg) divided by the squared height (m^2^)] [2,3], but indicating that the BMI based on self-reported anthropometric data is unreliable to define obesity in adults [2–4]. The degree to which these conclusions hold for adolescents is less clear. Adolescence is a very important period in life, characterized by physical and psychological evolutions [5]. Due to these large and rapid physical changes, self-reporting height and weight is less evident for adolescents compared to adults. Previous studies have shown good correlations between self-reported and measured height and weight in adolescents [6–13], but correlation coefficients are not the ideal measure of agreement [9]. Overall trends suggested systematic overestimation of height and underestimation of weight and BMI [7–16]. Important sources of bias were age, weight status, gender (inconsistent findings) and race [6–11,14–16]. The classification into BMI-categories using self-reported height and weight of the adolescents have not been shown as very accurate [8,10,11,14–17], except for two surveys [6,12].

The present study reconsiders the validity of self-reported height and weight of adolescents. The main objective is to investigate the accuracy of adolescents’ self-reported height and weight and the influence of using these self-reported values on the diagnosis of underweight, overweight and obesity, compared to the use of measured values. In addition, the study assesses the influence of their weighing behaviour on the accuracy of the adolescents’ self-reported BMI, based on the assumption that frequent weighing behaviour might influence the adolescents’ capability to estimate their weight and height more accurately.

## 2. Methods

### 2.1. Study Population

The study was performed in the region of Ghent, a medium sized city in Belgium. A sample of 10 to 18 year-old adolescents was drawn on the basis of a multistage cluster sampling technique. Ten secondary schools were randomly selected in the region of Ghent, using lists made available by the Flemish Ministry of Education and Training as sampling frame. All selected schools gave their permission to participate. When using formula for calculating differences between dependent populations [18], a sample size of 899, 796 and 538 was required for estimating a mean difference of 1 kg, 0.5 cm and 0.5 kg/m^2^ between reported and measured values with 95% probability for respectively weight, height and BMI. Because weight required the largest sample size (899 children), this parameter was used to determine our sample size goal. In the hope to reach a minimum of 100 children per school, the original sample size for schools was set at 10 secondary schools. However, during the first months of the fieldwork, response rate was calculated to allow additional sampling if necessary.

### 2.2. Questionnaire and Self-Reported Anthropometry

The adolescents were asked to fill in a questionnaire asking for their weight and height and questions concerning the frequency of measuring themselves at home and their eating patterns (whether they were following a diet or a special eating pattern like vegetarian). The adolescents were also categorized according to their educational level.

### 2.3. Anthropometric Measurements

This validation study was conducted in collaboration with the Centres for Pupils Counselling (‘Centrum voor Leerlingenbegeleiding’ or ‘CLB’). These CLBs are active in different domains of children’s and adolescents’ development and health [19]. In the context of preventive health care in children and adolescents, certain medical examinations are routinely performed, including weight and height measurements. The CLB staff is specifically trained to weigh and measure children and adolescents in a correct and standardized way (according to the protocol ‘VWVJ & Vlaamse Groeicurven’) [20]. A CLB nurse measured the adolescents’ weight and height when they were only wearing underwear. Weight was recorded to the nearest 0.1 kg, using an electronic balance (Seca 841) and height was measured to the nearest 0.1 cm, using a rigid stadiometer (Seca 220). The stadiometer was checked for accuracy and the balance was calibrated before examination. This standardized procedure was followed for all the adolescents in each local CLB.

### 2.4. Procedures

Adolescents routinely undergo an obligatory medical examination during the school year, carried out by a CLB nurse and doctor. Before entering the nursery room, the adolescents were given the questionnaire in which they were informed and invited to participate in the study, without mentioning that validation of anthropometric measurements was the objective of the study. They were asked to post the completed questionnaire and the signed informed consent in a box before entering the nursing room for the medical examination. Nurses of the CLBs were not allowed to read these forms or to open the box in order to prevent influencing by the self-reported weight and height of the adolescents. The study was carried out between September 2004 and June 2005. The Ethical Committee of the Ghent University Hospital granted ethical approval for the study.

### 2.5. Statistical Analysis

Self-reported and measured BMI (kg/m^2^) were calculated using self-reported and measured height and weight of the adolescents, respectively. Underweight, normal weight, overweight and obesity were identified by means of age- and gender-specific national and international cut-off points, which correspond with BMI values of 18.5, 25 and 30 kg/m^2^ at age 18 [20–22]. The overweight and obese categories are mutually exclusive, i.e., the overweight category does not include the obese. Paired-samples T tests were used to detect significant differences between measured and reported anthropometrics and Cohen’s d were calculated as effect size indices to evaluate the meaningfulness of the magnitude of the differences [23]. Cohen’s d values of ≤0.2, 0.21–0.79, and ≥0.8 indicate a small, medium, and large effect or meaning, respectively [23]. Intraclass correlation coefficients were calculated as a measure of association between measured and self-reported values [24]. Bland and Altman (B&A) plots were constructed to study agreement between self-reported and measured data on an individual level [25].

A misclassification analysis was performed to define the discordance between self-reported and measured BMI-categories. Differences in prevalence of underweight, overweight and obesity were obtained according to the Agresti method for comparing dependent proportions [26] and McNemar’s tests were performed to detect significant differences in these self-reported and measured prevalences [26]. The percentage of adolescents correctly classified, into an adjacent BMI-category classified, and grossly misclassified (in discrepant BMI-categories, e.g., self-reported as normal weight, while actually being obese) were calculated. The weighted kappa (*κ*) statistic was determined to study agreement between the self-reported and measured BMI-category (e.g., obese versus non obese), taking into account agreement by chance and the degree of disagreement [27,28]. Kappa values range between −1.00 (perfect disagreement) and 1.00 (perfect agreement), with a value of zero suggesting no agreement beyond chance alone. Kappa values less than 0.20 are considered as “poor” agreement, between 0.21 and 0.40 as “fair” agreement, between 0.41 and 0.60 as “moderate” agreement, between 0.61 and 0.80 as “good” agreement, and between 0.81 and 1.00 as “excellent” agreement [28]. The diagnostic value of self-reported weight and height to detect actual underweight, overweight and obesity in adolescents was also explored by the determination of sensitivity (the proportion of actual underweight, overweight or obese adolescents who are diagnosed correctly using the self-reported data), specificity (the proportion of adolescents who are not underweight, overweight or obese and are also not diagnosed as such using the self-reported data), positive predictive values (PPV: the proportion of adolescents diagnosed with underweight, overweight or obesity by means of self-reported data who also actually are) and negative predictive values (NPV: the proportion of adolescents not diagnosed with underweight, overweight or obesity by means of self-reported data and who actually are not) [29,30].

Proportional differences between self-reported and measured weight, height and BMI were calculated, using the following formula:

$$\begin{array}{c} (\text{Self-reported}\;\text{value}-\text{measured}\;\text{value})\times 100\\ \text{Measured}\;\text{value} \end{array}$$

One-Way ANOVA analyses were performed to investigate significant differences in mean proportional difference for BMI between the different categories of the demographic and weighing behaviour variables. To investigate whether the adolescents’ demographic characteristics and their weighing behaviour each accounted for an independent significant amount of variance in proportional differences between self-reported and measured anthropometrics, hierarchical regression analyses were performed. In four consecutive steps, the variables gender (with the females as reference), age (with the youngest age group of 10–12 year-olds as reference), educational level (with those from a secondary grammar or art school as a reference) and weighing behaviour (with those who weighed themselves during the past year as reference) were added to the model. Whether or not the adolescent weighed themselves during the past year was chosen as indicator of weighing behaviour in these models, since it most reflected the adolescents’ long-term weighing behaviour. The Statistical Package for the Social Sciences (SPSS) for Windows Version 15 was used for data management and statistical analyses. Unless reported differently, a P-value of 0.05 was used as threshold for statistical significance. Two-sided significance levels were quoted.

## 3. Results

### 3.1. Study Population

The sampling procedure yielded a sample of 1,014 adolescents, who were officially registered in the 70 sampled classes. Ten adolescents were non-eligible given their absence on the medical examination day in the CLB. Of the 1,004 eligible adolescents, 994 (99%) adolescents actually participated, of which 12 were excluded because they did not report their weight and/or height in the questionnaire. Subsequently, 982 adolescents were included in the validation. They had a mean age of 13.5 years (standard deviation (SD) + 1.4 yr) and 51.6% of them were boys. About 78.5% of the adolescents followed lessons on a secondary grammar or art school, while 18.3% followed a technical or vocational training (3.2% of the educational levels were missing). A total of 86.2% of the adolescents reported having a balance at home and 82.4% reported to have been weighing him- or herself during the past year. The percentage of adolescents weighing themselves daily, weekly or monthly were respectively 6.6%, 21.8% and 25.9%, while 44.5% reported to weigh themselves less than once a month. About 5.8% of the adolescents followed a diet (in 75.4% of the cases with the intention to lose weight) and a minority of 4% followed a specific food pattern like vegetarian or macrobiotic.

### 3.2. Agreement between Self-Reported and Measured Weight, Height and BMI

The self-reported and measured anthropometric data of the adolescents are presented in Table 1. Girls and boys both significantly underestimated their weight. However, girls significantly overestimated their height, in contrast to boys, where a significant underestimation was observed. The opposite biases for weight and height in girls resulted in a significant lower mean self-reported BMI compared to the mean actual BMI, while in boys no significant difference for BMI was observed. Cohen’s d are all below 0.20, indicating a small effect thus the magnitudes of the differences between both means are trivial.

**Figure 1.**
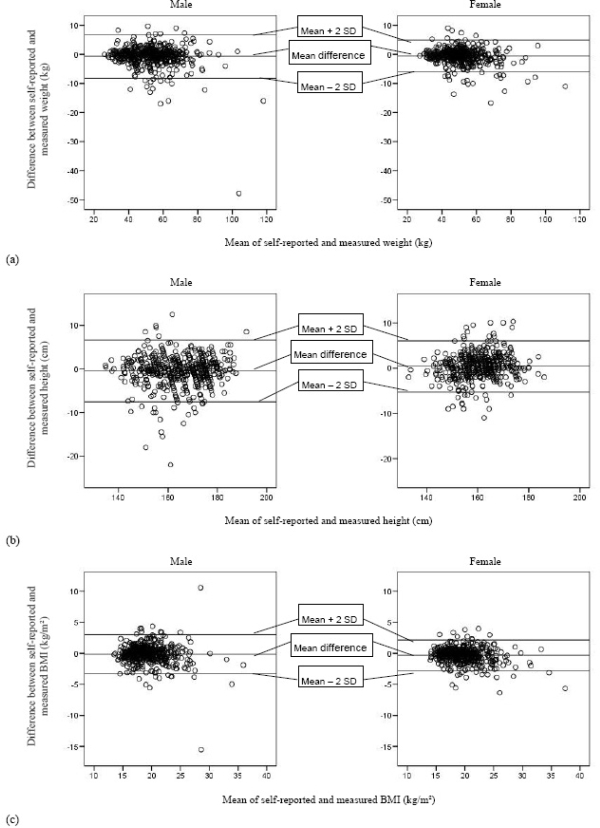
Bland and Altman plot of self-reported versus measured values of weight (a), height (b) and BMI (c) for boys (left) and girls (right).

Intraclass correlation coefficients between the self-reported and measured weight, height and BMI were respectively 0.961, 0.949 and 0.899 (all significant at the 0.01 level), indicating a high agreement between the self-reported and measured values. B&A plots [22] were also constructed to study agreement between self-reported and measured values on an individual level for boys and girls separately (see Figure 1).

The mean differences on the B&A plots are close to zero, indicating a rather good agreement between self-reported and measured values on population level. However, the ranges of misreporting of height, weight and BMI (indicated between the lines mean ±2SD) are quite large for both boys and girls and thus show limited agreement at individual level (see Figure 1).

### 3.3. Impact of Self-Reported Height and Weight on Classification in BMI-Categories

Prevalence of the self-reported and measured BMI-categories of the adolescents according to the national and international cut-off criteria are presented in Table 2.

For both national and international cut-offs, the 95% CI of the difference in prevalence of self-reported and measured underweight did not encompass value 0, indicating a statistically significant overestimation of the underweight prevalence when using self-reported height and weight. An underestimation of the prevalence of overweight and obesity was seen only for the international cut-offs. McNemar’s tests indicated significant differences in prevalences of underweight according to the national and international classification cut-offs, and in prevalences of overweight and obesity only according to the international classification cut-offs.

Table 3 shows the number and percentage of adolescents classified in the correct, adjacent or opposite BMI-category according to their self-reported BMI with their actual BMI as a standard.

For the national cut-offs, misclassification analysis showed that 849 (86.5%) adolescents were classified correctly, 130 (13.2%) were classified in the adjacent category, while only 3 (0.3%) were grossly misclassified. For the international cut-offs, these numbers were respectively 826 (84.1%), 153 (15.6%) and 3 (0.3%). The weighted kappa statistic to study agreement between self-reported and measured BMI-categories according to the national BMI cut-offs was 0.67 (95% CI = 0.59 to 0.75). For the international BMI cut-offs, a comparable weighted kappa of 0.63 (95% CI = 0.55 to 0.71) was found. These kappa values illustrated a ‘good agreement’ between the BMI-categories based on the self-reported and measured BMI. However, when using national and international cut-off values, respectively 25% and 32% of the adolescents actually being overweight or obese were diagnosed as being normal weight when using their self-reported values, while about half of the adolescents diagnosed with underweight are actually normal weight. If self-reported data were used, almost 5% of the total population would be missed for an intervention when aiming at overweight/obese adolescents, while about 2% of the total population would wrongly be encouraged to loose weight (according to international classification).

Table 4 summarizes the diagnostic values of self-reported height and weight to determine underweight, overweight and obesity. Sensitivity values (55.0–73.0%) for underweight, overweight and obesity were rather low, whereas specificity values (94.4–99.9%) and NPVs (94.7–99.4%) were very high, especially for the obesity category. PPVs were moderate for overweight and obesity (80.7–91.7%), but low for underweight (48.5–49.5%).

The weighted κ-values all indicated a good agreement for the classification of overweight and obesity based upon the self-reported BMI and according to international and national cut-offs. However, for the diagnosis of underweight, kappa values were slightly lower (0.50), indicating a moderate agreement for the classification of underweight between both methods (Table 4).

### 3.4. Possible Factors Associated with Bias in Self-Reported Weight, Height and BMI

The proportional difference in BMI according to the different categories of the demographic and weighing behaviour variables are shown in Table 5, together with the results from the One-Way ANOVA analyses.

Only for gender, a significant difference in mean proportional difference for BMI is present (*p* = 0.014). The self-reported BMI of adolescent boys reflected more closely their actual BMI compared to girls, given the smaller proportional difference for BMI. For educational level, the difference is borderline significant (*p* = 0.058) in favor of the higher educated from the secondary grammar or art school. For frequency of weighing ANOVA also showed a significant difference (*p* = 0.033), but Bonferroni Post-Hoc tests did not reveal any significant differences between the categories (data not shown). For the proportional difference between self-reported and measured height, results from ANOVA were similar as for BMI, indicating significant differences for gender and educational level (*p* < 0.001 for gender and *p* = 0.008 for educational level). However, it should be mentioned that for gender the difference was due to an effect of underreport of height in boys (proportional difference = −0.27) and an overreport in girls (proportional difference = +0.24), but the magnitude of misreporting was similar for both genders. For weight, only a significant difference in mean was seen for weighing behaviour, indicating that those who weighed themselves during the past year estimated their weight with a higher accuracy than those who did not (*p* = 0.003). Results from the hierarchical regression analysis to declare variance in proportional differences for BMI by means of demographic characteristics and weighing behaviour, is shown in Table 6.

Significant contributions on the variance in proportional difference between self-reported and measured BMI were observed for gender (step 1) and educational level (step 3). Similar results were observed for the variance in proportional difference for height (data not shown). For weight, the hierarchic model confirmed the importance of weighing behaviour by revealing a significant contribution of weighing behaviour to the variance in proportional difference for weight (data not shown).

## 4. Discussion

### 4.1. Principal Findings and Comparison with Previous Studies

This study showed that the self-reported height and weight of adolescents cannot replace the measured values for determining their BMI-category and thus measured weight and weight are highly recommended for the diagnosis of underweight, overweight and obesity. However, the adolescents as a group were actually quite capable of reporting their height and weight, despite the fact that their body undergoes large physical changes during the adolescent period of life. Although statistically significant differences arose between self-reported and measured weight, height and BMI (the latter only for girls) of the adolescents on group level, the magnitudes of these differences were trivial, given the low values for the effect size indices (Cohen’s d). However, B&A plots showed that these differences at individual level can be quite large, which indicated limited usefulness of the self-reported values on an individual level and for investigating the association with other health-related parameters. For height, the magnitude of the differences tended to slightly increase with increasing mean height, given the slightly divergent patterns in the B&A plots. This is also the case for BMI in boys, whereas in girls this divergent pattern is less present. Both boys and girls slightly, but significantly, underestimated their weight. This is in agreement with the findings of previous surveys [9–11,14]. However, other studies found that boys underestimated their weight to a lesser extent than girls did [6,7,12,13]. Elgar *et al.* and Abalkhail *et al.* reported only a significant underestimation of weight for girls [8,15]. The present study confirmed previous studies [7,9,11,13–15] with the finding that adolescent girls significantly overestimated their height, while only Strauss *et al.* [6] found a significant underestimation of height by girls. Adolescent boys in the present study significantly underestimated their height, which was reported previously by Strauss *et al.* [6], but in conflict with the previously reported overestimation in boys [7,11,13,14]. Other surveys found no significant over- or underestimation of height for both genders [8,10,12]. The present study showed only for girls a significant underestimation of BMI. This could be declared by the opposite biases in height (overestimation) and weight (underestimation) in girls. This effect is not seen in boys, where the biases are in the same direction (weight and height underestimated). Goodman *et al.* also reported an underestimation of BMI in girls, but they found a minor overestimation of BMI for boys [12]. Other surveys found for both genders an underestimation of BMI [7–11,13,17]. Correlations between self-reported and measured anthropometrics in the present study were rather high and comparable with results from previous studies [9–12], but slightly higher than those from others [6–8,13,16]. However, as mentioned before, the correlation coefficient is not an ideal measure for agreement, since systematic over- or underreports cannot be taken into account. A high correlation does not automatically imply a good agreement between two measurements.

When using the adolescents’ self-reported anthropometrics for classification in BMI-categories according to the national and international cut-off points, respectively 86.5% and 84.1% of the adolescents were correctly classified in the present study. Some misclassification errors occurred, in general leading to a statistically significant underestimation of the prevalence of overweight and obesity (only according to the international criteria), as also reported by Elgar *et al.* [8] and Tokmakidis *et al.* [11], and overestimation of the prevalence of underweight, the latter only being found by Danubio and colleagues in young adult girls (18–35 years) [17]. The relative magnitudes of these under- (15.5% for overweight and 41% for obesity) and overestimations (24.7% for underweight) are indicative of a substantial significance on clinical or public health level. The conclusions for overweight are in line with those made by Sherry and colleagues, who made a literature review on this topic of studies performed in the United States [31]. The overestimation of underweight in adolescents in the present study is, to our knowledge, new information. Weighted κ-values (≥0.63) illustrated a good statistical agreement between the self-reported and measured BMI-categories for both national and international cut-offs. However, the sensitivity and specificity values and PVs of self-reported anthropometrics for predicting underweight, overweight and obesity questioned the diagnostic value of self-reported anthropometrics to identify actual underweight, overweight and obesity in epidemiological surveys. Previous studies reported similar values for sensitivity and specificity as in the present study [8,12,15]. However, direct comparison is not always feasible since other cut-off criteria for the BMI-categories were implemented. In general, classification according to the national criteria resulted in higher sensitivity and specificity values, indicative for a better classification. A possible explanation for this finding could be the higher agreement between the study sample and the population on which the national cut-off criteria are based on. When comparing the classification of adolescents in BMI categories based on their self-reported and measured BMI, at least 25% of the adolescents who should be targeted for nutritional advice on base of their actual overweight/obese status would be missed for intervention purposes when using self-reported weight and height values. Conversely, about half of the adolescents being classified as underweight when using self-reported data could be wrongly encouraged to gain weight. From those results one can conclude that an important number of adolescents within the target group would be missed for interventions, while almost half of the adolescents who would get an intervention for gaining weight won’t need this intervention. Therefore, self-reported weight and height should not be used when aiming at classifying adolescents for intervention purposes.

The present study demonstrated that boys and the adolescents with a higher educational level had a higher accuracy of their self-reported BMI, compared to girls and those with a lower educational level respectively. This gender difference again illustrates the strengthening effect of opposite biases in weight and height for girls, as mentioned above, resulting in a lower accuracy of self-reported BMI. The finding that girls underestimated their weight and overestimated their height could be explained by the fact that they want to meet the social desirability to be thin and tall, but further investigation is needed to confirm this assumption. The difference across educational levels is somewhat more complex and possible explanations could be the lack of interest or the lower capability to estimate their height in the adolescents from a lower educational level. Further research is necessary to investigate these assumptions. The accuracy of the self-reported weight was only positively influenced by actual weighing during the last year and not by educational level nor gender. Elgar *et al.* also did not find an influence of gender on the accuracy of self-reported weight, but they found that self-perceptions of body size predicted bias in self-reported weight [8]. This could not be investigated in the present study. The effect of weighing behaviour on the accuracy of self-reported weight should be considered when planning self-reported anthropometric assessments in future surveys where measuring height and weight of adolescents is not feasible. One could give the guideline to the adolescents weigh themselves at home before completing the survey in order to obtain a more accurate self-reported weight. However, to our knowledge, little is known about the guideline compliance of adolescents in this context. The lack of good-quality equipment at home could also give a wrong indication of their weight. Future research should investigate the effect of above-mentioned guideline and the influence of measurement conditions at home on the accuracy self-reporting.

Sherry *et al.* gave the guideline to examine the feasibility of measuring height and weight on a community level [31]. This was not a general aim of the present study, but the collaboration with the CLBs was very successful and a proof that good-quality assessments of height and weight on a large scale are indeed feasible. Future obesity-related investigations are encouraged to collaborate with the national institutions which are responsible for routine screening of the clinical health of adolescent populations.

### 4.2. Strengths and Limitations

One of the major strengths of the present study is the large sample size, encompassing both boys and girls from different educational levels and with a participation rate of 99%. Also noteworthy is the large level of standardization of the process by working via the CLBs with trained nurses, and with a maximum time interval of one-hour between self-reporting and measuring of weight and height. The study was performed in adolescents from Ghent and thus the results cannot be generalized to all Belgian adolescents. Still, they give a good indication of the present situation in adolescents from the region of Ghent, given the large study sample and the broad age range.

## 5. Conclusions

The adolescents were in general quite capable to report their own height and weight with good accuracy. However, using the adolescents’ self-reported anthropometric values to determine their BMI-category lead to an underestimation of overweight and obesity and overestimation of underweight. The degree of misclassification errors and the diagnostic value of the self-reported anthropometrics of the adolescents in the present study are in favor of using measured anthropometrics for the diagnosis of underweight, overweight and obesity in practice and future large-scale epidemiological surveys and interventions. If it is only feasible, for instance for logistic reasons, to obtain self-reported anthropometrics, it could be recommended to give the guideline to the adolescents to weigh themselves before participating in the survey, to improve the accuracy of their self-reported weight.

## Figures and Tables

**Table 1.** Comparison of self-reported and measured weight, height and BMI among 982 adolescents.

	Self-reported		Measured		Difference in Mean		*P*^b^	*d^c^*

	Mean (SD)		Mean (SD)		(95% CI)^a^			

Girls (n = 475)								
Weight (kg)	50.9	(10.8)	51.5	(11.4)	−0.60	(−0.85 to −0.36)	<0.001	0.05
Height (cm)	160.9	(8.7)	160.5	(8.2)	0.38	(0.13 to 0.64)	0.004	0.05
BMI (kg/m^2^)	19.5	(3.1)	19.9	(3.4)	−0.32	(−0.43 to −0.20)	<0.001	0.12

Boys (n = 507)								
Weight (kg)	52.6	(12.5)	53.3	(13.2)	−0.67	(−1.00 to −0.34)	<0.001	0.06
Height (cm)	164.1	(11.2)	164.6	(11.1)	−0.47	(−0.78 to −0.16)	0.003	0.05
BMI (kg/m^2^)	19.3	(3.0)	19.4	(3.2)	−0.12	(−0.25 to 0.02)	0.098	0.03

^a^ 95% CI = 95% confidence interval

^b^ According to the paired-samples T-test

^c^ *d = Cohen’s d* values calculated as effect size index

**Table 2.** Categorization of the adolescents (n = 982) in different BMI-categories according to national [20] and international [21,22] classification cut-off points, and differences between self-reported and measured prevalence in each category.

	Self-reported		Measured		Difference^a^		*P^b^*

	% (n)		% (n)		% (95% CI)		

Underweight (Nat. Class.)	9.3	(91)	7.4	(73)	1.83	(0.12 to 3.55)	0.048
Underweight (Int. Class.)	10.1	(99)	7.9	(78)	2.14	(0.35 to 3.93)	0.026
Normal weight (Nat. Class.)	77.9	(765)	78.1	(767)	−0.20	(−2.46 to 2.05)	0.930
Normal weight (Int. Class.)	77.5	(761)	76.8	(754)	0.71	(−1.72 to 3.15)	0.623
Overweight (Nat. Class.)	11.6	(114)	12.8	(126)	−1.22	(−2.71 to 0.27)	0.142
Overweight (Int. Class.)	11.2	(110)	13.2	(130)	−2.04	(−3.73 to −0.35)	0.025
Obese (Nat. Class.)	1.2	(12)	1.6	(16)	−0.41	(−.97 to 0.16)	0.289
Obese (Int. Class.)	1.2	(12)	2.0	(20)	−0.82	(−1.44 to −0.19)	0.021

^a^ Difference (+95% CI) in prevalence of underweight, overweight and obesity were obtained according to the Agresti method for comparing dependent proportions

^b^ According to McNemar’s test

**Table 3.** The number and proportion of adolescents (n = 982) classified in different BMI categories according to the self-reported and measured values of height and weight, using a) national classification cut-off values [20]; b) international classification cut-off values [21,22].

a) National classification cut-off values											
		BMI-category based on MEASURED BMI								Total n (%)	
		Underweight n (%)		Normal weight n (%)		Overweight n (%)		Obese n (%)			

BMI-category based on SELF-REPORTED BMI	Underweight	45	(4.6)	46	(4.7)	0	(0.0)	0	(0.0)	91	(9.3)
	Normal weight	28	(2.9)	702	(71.5)	33	(3.4)	2	(0.2)	765	(77.9)
	Overweight	0	(0.0)	18	(1.8)	92	(9.4)	4	(0.4)	114	(11.6)
	Obese	0	(0.0)	1	(0.1)	1	(0.1)	10	(1.0)	12	(1.2)

	Total	73	(7.4)	767	(78.1)	126	(12.8)	16	(1.6)	982	(100)

b) International classification cut-off values											
		BMI-category based on MEASURED BMI								Total n (%)	
		Underweight n (%)		Normal weight n (%)		Overweight n (%)		Obese n (%)			

BMI-category based on SELF-REPORTED BMI	Underweight	48	(4.9)	51	(5.2)	0	(0.0)	0	(0.0)	99	(10.1)
	Normal weight	30	(3.1)	683	(69.6)	46	(4.7)	2	(0.2)	761	(77.5)
	Overweight	0	(0.0)	19	(1.9)	84	(8.6)	7	(0.7)	110	(11.2)
	Obese	0	(0.0)	1	(0.1)	0	(0.0)	11	(1.1)	12	(1.2)

	Total	78	(7.9)	754	(76.8)	130	(13.2)	20	(2.0)	982	(100)

**Table 4.** Diagnostic properties of self-reported height and weight for the diagnosis of underweight, overweight and obesity in adolescents (n = 982).

		Sensitivity		Specificity		PPV^a^		NPV^b^		Kappa statistic	

BMI categorie		%	(95% CI)	%	(95% CI)	%	(95% CI)	%	(95% CI)	κ	(95% CI)

National classification [20]											
	Underweight	61.6	(50.2 to 72.0)	94.9	(93.3 to 96.2)	49.5	(38.3 to 60.7)	96.9	(95.5 to 97.8)	0.51	(0.36 to 0.66)
	Overweight	73.0	(64.7 to 80.0)	97.4	(96.1 to 98.3)	80.7	(72.9 to 86.6)	96.1	(94.6 to 97.2)	0.73	(0.61 to 0.85)
	Obese	62.5	(38.6 to 81.5)	99.8	(99.3 to 99.9)	83.3	(59.3 to 94.5)	99.4	(98.7 to 99.7)	0.71	(0.34 to 1.00)

International classification [21,22]											
	Underweight	61.5	(50.4 to 71.6)	94.4	(92.7 to 95.7)	48.5	(37.8 to 59.4)	96.6	(95.2 to 97.6)	0.50	(0.36 to 0.64)
	Overweight	64.6	(56.1 to 72.3)	97.0	(95.6 to 97.9)	76.4	(68.4 to 82.8)	94.7	(93.0 to 96.0)	0.66	(0.54 to 0.78)
	Obese	55.0	(34.2 to 74.2)	99.9	(99.4 to 99.9)	91.7	(72.0 to 97.9)	99.1	(98.2 to 99.5)	0.68	(0.34 to 1.00)

^a^ PPV = Positive Predictive Value;

^b^ NPV = Negative Predictive Value

**Table 5.** Proportional differences between self-reported and measured BMI of adolescents according to demographic and weighing behaviour characteristics.

	Difference in BMI^a^		P^b^
Characteristic	Mean	(SD)	
Total	− 0.75	(6.66)	
Gender			0.014
Male	−0.24	(7.27)	
Female	−1.29	(5.89)	
Age			0.656
10–12 yrs	−0.68	(6.85)	
13–18 yrs	−0.89	(6.24)	
Educational level			0.058
Secondary grammar or art school	−0.60	(6.36)	
Technical or vocational training	−1.62	(6.95)	
Self weighed during the past year			0.122
Yes	−1.48	(9.46)	
No	−0.61	(5.92)	
Balance at home			0.968
Yes	−0.75	(6.14)	
No	−0.72	(9.36)	
Frequency of weighing			0.033
Daily	−1.34	(5.71)	
Weekly	−0.09	(4.77)	
Monthly	−1.69	(6.01)	
Less than once a month	−0.45	(7.70)	
Following a diet			0.098
Yes	−2.17	(9.40)	
No	−0.67	(6.65)	
Having a specific food pattern			0.352
Yes	−1.73	(7.36)	
No	−0.71	(6.63)	

^a^ This difference is the proportional difference between the self-reported and measured BMI ((Self-reported BMI - measured BMI) / self-reported BMI × 100)

^b^ According to One-Way ANOVA analysis.

**Table 6.** Hierarchical regression models for predicting proportional differences between the adolescents’ self-reported and measured BMI by means of demographic characteristics and weighing behaviour.

	STEP 1			STEP 2			STEP 3			STEP 4		
Variable	B	SE B	β	B	SE B	β	B	SE B	β	B	SE B	β
Gender	−1.06	0.42	−0.082*	−1.06	0.42	−0.082*	−1.05	0.42	−0.081*	−1.09	0.42	−0.084*
Age				−0.15	0.45	−0.011	−0.42	0.47	−0.030	−0.43	0.47	−0.031
Educational level							1.15	0.56	0.069*	1.09	0.56	0.066
Weighing behaviour										0.72	0.56	−0.042
R^2^ change	0.007*			0.000			0.004*			0.002		

* *P* < 0.05,

** *P* < 0.01,

*** *P* < 0.001
